# Dose-Dependent Effects of Endotoxin on Neurobehavioral Functions in Humans

**DOI:** 10.1371/journal.pone.0028330

**Published:** 2011-12-02

**Authors:** Jan-Sebastian Grigoleit, Jennifer S. Kullmann, Oliver T. Wolf, Florian Hammes, Alexander Wegner, Stephanie Jablonowski, Harald Engler, Elke Gizewski, Reiner Oberbeck, Manfred Schedlowski

**Affiliations:** 1 Institute of Medical Psychology and Behavioral Immunobiology, University of Duisburg-Essen, Essen, Germany; 2 Department of Diagnostic and Interventional Radiology and Neuroradiology, University Hospital Essen, Essen, Germany; 3 Department of Cognitive Psychology, Ruhr-University Bochum, Bochum, Germany; 4 Department of Trauma Surgery, University Hospital Essen, Essen, Germany; University of North Dakota, United States of America

## Abstract

Clinical and experimental evidence document that inflammation and increased peripheral cytokine levels are associated with depression-like symptoms and neuropsychological disturbances in humans. However, it remains unclear whether and to what extent cognitive functions like memory and attention are affected by and related to the dose of the inflammatory stimulus. Thus, in a cross-over, double-blind, experimental approach, healthy male volunteers were administered with either placebo or bacterial lipopolysaccharide (LPS) at doses of 0.4 (*n* = 18) or 0.8 ng/kg of body weight (*n* = 16). Pro- and anti-inflammatory cytokines, norephinephrine and cortisol concentrations were analyzed before and 1, 1.75, 3, 4, 6, and 24 h after injection. In addition, changes in mood and anxiety levels were determined together with working memory (n-back task) and long term memory performance (recall of emotional and neutral pictures of the International Affective Picture System). Endotoxin administration caused a profound transient physiological response with dose-related elevations in body temperature and heart rate, increases in plasma interleukin (IL)-6, IL-10, tumor necrosis factor (TNF)-α and IL-1 receptor antagonist (IL-1ra), salivary and plasma cortisol, and plasma norepinephrine. These changes were accompanied by dose-related decreased mood and increased anxiety levels. LPS administration did not affect accuracy in working memory performance but improved reaction time in the *high-dose* LPS condition compared to the control conditon. In contrast, long-term memory performance was impaired selectively for emotional stimuli after administration of the lower but not of the higher dose of LPS. These data suggest the existence of at least two counter-acting mechanisms, one promoting and one inhibiting cognitive performance during acute systemic inflammation.

## Introduction

The release of pro-inflammatory cytokines during infection and inflammation can affect behavior, mood, and functioning of the central nervous system (CNS) [1], [2], [3]. Behavioral changes induced by immune activation include symptoms such as psychomotor slowing, social withdrawal, anhedonia, depressed mood, and disturbed sleep architecture, collectively termed “sickness behavior” [4], [5], [6]. In addition, there is growing experimental and clinical evidence implicating systemic inflammation to be involved in the pathophysiology of neuropsychiatric diseases such as depression and schizophrenia as well as age-related cognitive decline [7], [8], [9], [10], [11], [12], [13], [14]. Animal and human studies suggest that pro-inflammatory cytokines such as interleukin (IL)-1, IL-6, and tumor necrosis factor (TNF)-α play a pivotal role in mediating sickness-related behavior and cognitive impairments by communicating peripheral inflammation to the brain [1], [3], [15], [16], [17]. Studies in experimental animals demonstrated that systemic immune activation by viral, bacterial, or parasitic infections result in impaired memory functioning [18], [19], [20], [21], [22], [23]. Experimental approaches in humans, employing lipopolysaccharide (LPS)-induced immune activation to investigate the effects of a peripheral inflammatory response on learning and memory reported either increased [24], no [25], [26], [27] or decreased [28] cognitive performance after LPS administration. Reasons for these discrepancy might be the different quality and concentrations of LPS administered together with distinct time intervals of testing memory performance and the different memory processes analyzed [25].

**Figure 1:**
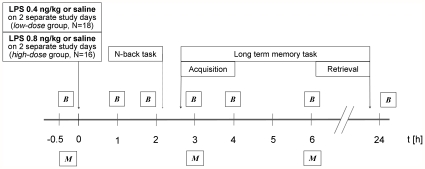
Experimental design. Volunteers arrived 1 h prior to the experiment. Thirty minutes before the injection, an intravenous cannula was inserted into the antecubital forearm vein for intermittent blood sampling and drug injection. Before and 1, 1.75, 3, 4, and 6 h after the injection blood samples (B) were collected and vital parameters were assessed. Before as well as 3 and 6 h after the injection the subjects completed mood and anxiety questionnaires (M). After the 1.75 h blood collection the n-back task was performed, and before the 3 h collection the acquisition for the long-term memory test took place. When showing no signs of inflammation or any other kind of illness, subjects were discharged and returned on the following day for follow-up examination and completing the long-term memory task. The whole procedure was repeated on another study day with a 10–14 days delay. Every participant received the higher or lower dose of LPS during one and saline during the other test session in a balanced, randomized manner.

Therefore, the current study analyzed memory performance before and after administration of two different dosages of LPS in healthy humans in a double blinded, cross-over, placebo-controlled design (Fig. 1). Subjects either received an injection of 0.4 ng/kg (*n* = 18) or 0.8 ng/kg (*n* = 16) LPS or placebo, respectively, and were tested for working memory and long-term memory performance. Furthermore, self reported mood, attention, and anxiety was assessed. Blood samples were drawn before and 1, 1.75, 3, 4, 6, and 24 hours after LPS or placebo administration and analyzed for plasma levels of pro- and anti-inflammatory cytokines as well as cortisol and norepinephrine concentrations.

## Materials and Methods

### Ethics Statement

The study was approved by the local ethics committee of the University of Duisburg-Essen and follows the rules stated in the Declaration of Helsinki. Study participants were informed about the study design, and were only enrolled in the experiment after written informed consent had been obtained.

### Subjects

Thirty-four male subjects participated in the study. They were randomly allocated to one of the two experimental groups (see below). All subjects underwent an extensive physical and psychiatric screening. The physical examination included a complete blood cell count, liver enzymes, renal parameters, electrolytes, coagulation factors, and C-reactive protein (CRP). An interview was conducted to exclude the presence or history of any physical or psychiatric disorder. The groups did not differ in age, years of education or body weight, nor in any of the physical or psychological screening parameters, and there was no detectable influence of these parameters on any of the outcome measures. Subjects and investigators where blinded with respect to the study condition.

### Experimental design

In this placebo-controlled, double-blind, crossover study, participants were randomly allocated to two different groups: the *high-dose* group (*n* = 16; mean age: 25.3±3.3 years; range: 19–29 years; mean body mass index (BMI): 23.0±2.5; range: 19.4–27.2) received a dose of 0.8 ng LPS per kg of body weight on one occasion and placebo (0.9% saline) on another occasion in balanced order; in the *low-dose* group (*n* = 18; mean age: 26.4±3.2 years; range: 20–33 years; BMI: 24.6±2.6; range: 20.3–30.5), subjects received 0.4 ng LPS per kg and placebo on two different occasions. Between the two experimental conditions was a rest period of 10 to 14 days. The experiments were conducted in a medically equipped room and were supervised by emergency physicians of the Department of Trauma Surgery. An intravenous cannula was inserted into the antecubital forearm vein for intermittent blood sampling and drug injection. Saline and LPS injections were always performed around noon. The endotoxin (*Escherichia coli*, Lot G3E069, United States Pharmacopeia, Rockville, Maryland, USA) had been prepared for use in humans. Briefly, the lyophilized LPS was dissolved in sterile water, filtered through a 0.2-µm membrane, and subjected to a microbial safety testing routine approved by the German Federal Agency for Sera and Vaccines (Paul-Ehrlich Institute, Langen, Germany). The LPS solution was stored in endotoxin-free borosilicate tubes at −20°C until use. During the experimental sessions, the subjects completed questionnaires concerning their mental state and performed several neuropsychological tests (see below). Blood and saliva samples were drawn at baseline and 1, 1.75, 3, 4, 6, and 24 h post-injection. Plasma for the measurement of cytokine, cortisol, and norepinephrine levels was separated by centrifugation and stored at −80°C until analysis. Temperature, heart rate, and blood pressure were analyzed immediately prior to the blood collections using an aural thermometer and a blood pressure cuff. At the end of each experimental day, subjects underwent a physical examination before being discharged from the clinical research unit and returned for thorough follow-up examinations 24 h and 1 week after the experimental session.

### Cytokine and hormone determinations

Plasma cytokine concentrations were quantified using multiplexed bead-based assays (Bio-Plex Cytokine Assays, Bio-Rad Laboratories GmbH, Munich, Germany). Samples were prepared according to the manufacturer's instructions and were analyzed on a triple-laser FACSCanto II flow cytometer using FACSDiva software (BD Immunocytometry Systems, Heidelberg, Germany). Absolute cytokine levels were calculated based on the mean fluorescence intensity of cytokine standard dilutions with a 4 Parameter Logistics (4PL) curve model using GraphPad Prism 5 (GraphPad Software Inc., La Jolla, CA, USA). The detection limit of the assays was 60 pg/ml for IL-1ra, 0.2 pg/ml for IL-6, 0.4 pg/ml for IL-10, and 3 pg/ml for TNF-α. Plasma levels of cortisol and norepinephrine and salivary levels of cortisol were determined using enzyme-linked immunosorbent assays (ELISA; Labor Diagnostika Nord, Nordhorn, Germany [norepinephrine] and IBL International, Hamburg, Germany [cortisol]) according to the test protocol of the manufacturer, and were analyzed on a Fluostar OPTIMA Microplate Reader (BMG Labtech, Offenbach, Germany). The detection limits were 50 pg/ml (norepinephrine) and 0.83 nmol/l (cortisol), respectively.

### Mood and Anxiety Questionnaires

Subjects had to complete two standardized self-report questionnaires immediately before as well as 3 and 6 h after LPS or saline injection: 1. The multidimensional mood questionnaire (Multidimensionaler Befindlichkeitsfragebogen, MDBF), which is a well-established and -validated scale in German language. The MDBF provides sub-scales for positive vs. negative mood, alertness vs. fatigue and calmness vs. uneasiness [29], [30]. 2. The State Trait Anxiety Inventory (STAI, state version), a widely used scale to quantify anxiety in health research [31], [32]. The STAI consists of twenty items with four answer choices each.

### Memory tests

Between the blood withdrawals at 1.75 and 3 h, the n-back task (2-back version) for working memory performance and the first part of the long-term memory task took place. The n-back task is a common and well-established continuous performance test for measurements of working memory performance [33], [34]. After a training block, which was not assessed, a sequence of 155 letters, divided into 5 blocks of 31 letters was presented on a screen. Within a block each letter was presented for 1 second directly followed by the next. After each block there was a break of 30 seconds. The subjects were told to press a button whenever the current letter shown was the same as the last but one (Fig. S2). Overall the test included 25 of those adequate stimuli, which were distributed randomly over the entire sequence. The software (n-Back fmri v0.93 by Frank Schulte used on a Sony Vaio laptop computer) allows assessing the number of false and correct reactions as well as the reaction time that is needed to press the button after the appearance of an adequate stimulus. To evaluate the subjects' working memory performance, accuracy was calculated by subtracting the number of reactions to inadequate stimuli (false alarms) from the number of correct reactions to the adequate stimuli.

In order to analyze long-term memory performance, subjects were presented a randomized sequence of 72 pictures from the International Affective Picture System (IAPS, see Fig. S1Xfor examples). The images were presented back to back for three seconds, respectively on a laptop computer screen. Whereas 50% of pictures had neutral contents and were reported to elicit medium levels of valence and low levels of arousal, 50% of pictures consisted of highly emotional pictures known to elicit high arousal and low valence values [35]. 24 h after the administration of LPS or placebo, respectively, participants were presented a second sequence of 40 pictures containing 20 of those they were presented the day before and 20 new ones of similar content, again with 50% of emotional and 50% of neutral content, respectively. Subjects were asked to indicate every recognized picture. For evaluation of memory performance accuracy was calculated by subtracting the number of new pictures remembered by mistake (false alarms) from the number of correctly recognized stimuli (correct hits) for every subject. Since all subjects participated in two experimental days (receiving LPS or saline as a control condition) we employed two counter-balanced, randomly applied sets of IAPS pictures.

### Statistics

Statistical analyses were performed using SPSS 17 (SPSS Inc., Chicago, IL) and GraphPad Prism 5 (GraphPad Software, Inc., San Diego, CA). For the analyses of temperature, heart rate, cytokines, hormones, and mood parameters differences between experimental and control condition were analyzed using repeated measures analysis of variance (ANOVA) with ‘treatment’ as between-subjects factor (LPS/placebo) and ‘time’ as repeated measures within-subject factor. In case of a significant time×treatment interaction, Bonferroni post hoc tests were computed to assess mean differences between the control and endotoxin condition for specific time points. Absolute changes in body temperature, heart rate, cytokine concentrations, hormone concentrations, and mood following endotoxin administration were calculated by subtracting the control values of each individual from the corresponding values of the experimental condition. To compare those changes between the *low-dose* and the *high-dose* groups, repeated measures ANOVA followed by Bonferroni post hoc test was used. Differences in the n-back task for working memory were calculated using ANOVA with group as between subjects and treatment as within subject factor followed by Bonferroni post hoc tests. Differences in the long term memory task were computed by ANOVA with group as between subject factor and treatment and stimulus-quality (emotionality) as between-subject factors, followed by Bonferroni corrected paired and unpaired t-tests. In all statistical analyses *p*<0.05 was considered significant.

Most of the data reported here were normally distributed (Kolmogorov-Smirnov and the D'Agostino and Pearson omnibus normality test). We observed non-normal distributions for TNF-α levels (4–24 h, both groups), IL-6 levels (4 h, *high-dose* group), IL-1ra levels (1.75 h, both groups), and saliva cortisol levels (*high-dose* group at 4 and 24 h). Psychological variables showed non-normal distributions for calmness (3 h and 6 h) and anxiety (3 h). In contrast, all memory data were normally distributed. However, re-calculation with log-transformed data to restore normal distribution confirmed the reported results.

Pearson correlation analyses were performed to analyze possible associations between the neuropsychological outcome measures and the immunological, physiological, and neuroendocrine parameters. Changes in neuropsychological and mood variables which were significantly affected by the endotoxin treatment were correlated with changes in cytokines, neuroendocrine, and vital parameters. The alpha level was adjusted according to the respective number of comparisons (Bonferroni correction).

## Results

### Body temperature and heart rate

**Figure 2:**
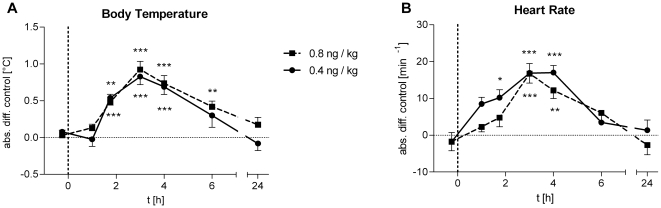
Body temperature and heart rate. Absolute changes in body temperature and heart rate following the administration of 0.4 (solid lines, *n* = 18) or 0.8 ng/kg *E. coli* endotoxin (dashed lines, *n* = 16). Data are presented as means ± SEM. Significant differences between experimental condition and respective saline control: *p<0.05, **p<0.01, ***p<0.001 (Bonferroni post hoc test).

Both, *low-dose* and *high-dose* LPS treatment significantly increased body temperature (Fig. 2A; *low-dose*: *F* = 13.18, *p<*0.001; *high-dose*: *F* = 14.11, *p<*0.001) and heart rate (Fig. 2B; *low-dose*: *F* = 10.49, *p*<0.001, *high-dose*: *F* = 9.12, *p*<0.001) without significant differences between LPS doses.

### Cytokine levels

**Figure 3:**
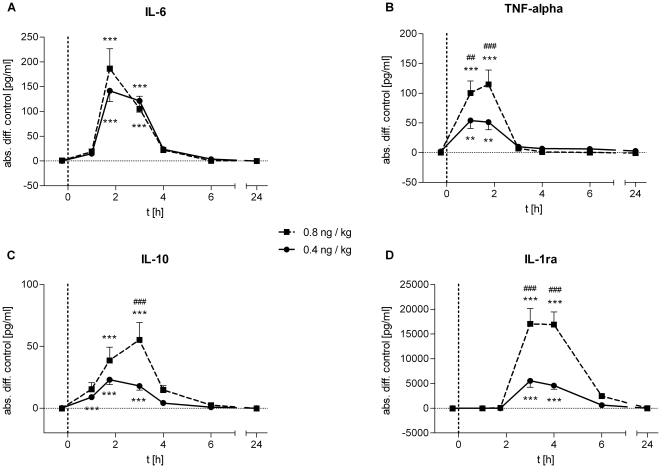
Cytokine response to LPS administration. Absolute changes in plasma concentrations of pro- (IL-6, TNF-α) and anti-inflammatory (IL-10, IL-1ra) cytokines after administration of 0.4 (solid lines, *n* = 18) or 0.8 ng/kg *E. coli* endotoxin (dashed lines, *n* = 16). Data are presented as means ± SEM. Significant differences between experimental condition and respective saline control: *p<0.05, **p<0.01, ***p<0.001; significant differences between changes in *high*- and *low-dose* condition: ##*p*<0.01, ###*p*<0.001 (Bonferroni post hoc test).

Endotoxin administration induced significant increases in plasma concentrations of the pro-inflammatory cytokines IL-6 (*low-dose*: *F* = 31.11, *p*<0.001, *high-dose*: *F* = 17.74, *p*<0.001) and TNF-α (*low-dose*: *F* = 12.05, *p*<0.001, *high-dose*: *F* = 21.26, *p*<0.001) with most pronounced increases 1.75 h after LPS injection (Fig. 3). Plasma TNF-α levels were significantly higher in the *high-dose* compared to the *low-dose* group (*F* = 5.02, *p*<0.001; Fig. 3B), whereas IL-6 levels did not significantly differ between doses (Fig. 3A). The anti-inflammatory cytokine IL-10 was significantly increased in both endotoxin groups (*low-dose*: *F* = 25.47, *p*<0.001; *high-dose*: *F* = 12.80, *p*<0.001) with significantly more pronounced elevations in the *high-dose* group 3 h post injection (*F* = 5.02, *p*<0.001; Fig. 3C). The most pronounced differences in cytokine levels between the two groups were observed for IL-1 receptor antagonist (IL-1ra) *(F* = 15.59, *p*<0.001, *low-dose* vs. *high-dose; F* = 33.45, *p*<0.001, *high-dose* vs. control*; F* = 21.01, *p<.001*, *low-dose* vs. control; Fig. 3D).

### Neuroendocrine measures

**Figure 4:**
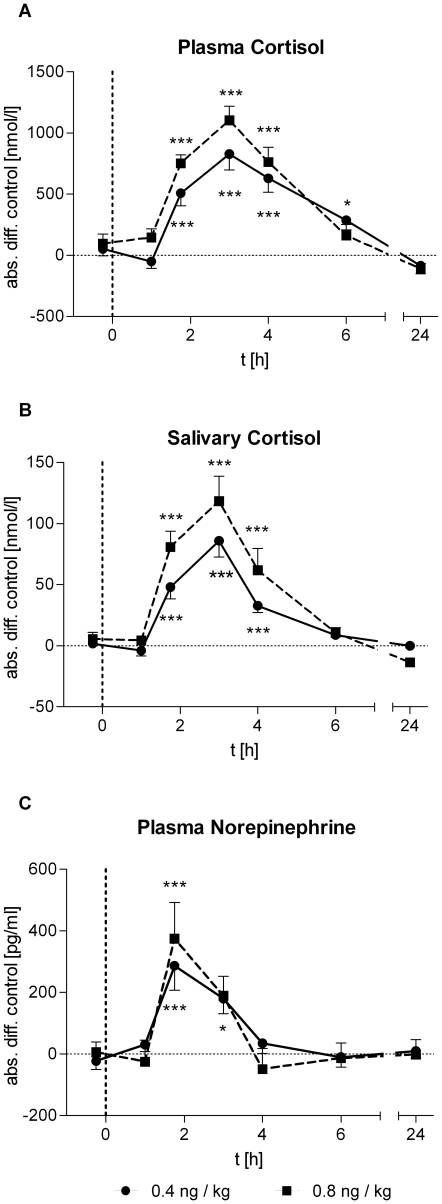
Neuroendocrine responses to LPS administration. Absolute changes in plasma concentrations of cortisol and norepinephrine and salivary cortisol levels after administration of 0.4 (solid lines, *n* = 18) or 0.8 ng/kg *E. coli* endotoxin (dashed lines, *n* = 16). Data are presented as means ± SEM. Significant differences between experimental condition and respective saline control: *p<0.05, **p<0.01, ***p<0.001 (Bonferroni post hoc test).

Activation of the innate immune response by endotoxin increases the activity of HPA axis and sympathetic nervous system. Thus, we analyzed plasma and saliva cortisol concentrations as well as plasma norepinephrine levels. The neuroendocrine response to LPS injection was reflected by marked increases in the levels of total cortisol in plasma (*low-dose*: *F* = 19.44, *p*<0.001; *high-dose*: *F* = 29.99, *p*<0.001; Fig. 4A) and free cortisol in the saliva (*low-dose*: *F* = 24.80, *p*<0.001; *high-dose*: *F* = 20.40, *p*<0.001; Fig. 4B) as well as plasma norepinephrine (*low-dose*: *F* = 8.12, *p*<0.001; *high-dose*: *F* = 5.17, *p*<0.001; Fig. 4C). The rise of free cortisol in saliva was significantly higher in the *high-dose* group than in the *low-dose* group (*F* = 2.27, *p*<0.05).

### Mood & Anxiety

**Figure 5:**
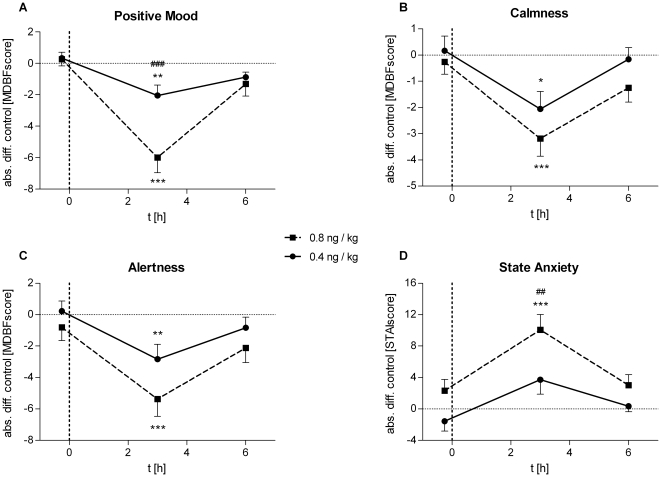
LPS effects on mood and anxiety. Absolute changes in mood, calmness, and alertness (MDBF) and in state anxiety (STAI) after administration of 0.4 (solid lines, *n* = 18) or 0.8 ng/kg *E. coli* endotoxin (dashed lines, *n* = 16). Data are presented as means ± SEM. Significant differences between experimental condition and respective saline control: *p<0.05, **p<0.01, ***p<0.001; significant differences between changes in *high-* and *low-dose* condition: ##*p*<0.01, ###*p*<0.001 (Bonferroni post hoc test).

The effects of endotoxin treatment on mood and anxiety were analyzed with two standardized questionnaires (MDBF, STAI) at baseline as well as 3 h and 6 h after LPS injection (Fig. 5). Self-reported positive mood (*low-dose*: *F* = 6.21, *p* = 0.01; *high-dose*: *F* = 25.97, *p*<0.001), calmness (*low-dose*: *F* = 6.42, *p*<0.01; *high-dose*: *F* = 5.52, *p*<0.01), and alertness (*low-dose*: *F* = 5.07, *p*<0.01; *high-dose*: *F* = 7.09, *p*<0.01), were significantly decreased 3 h after endotoxin injection in both groups. The decrease in positive mood was significantly more pronounced (−6.00 vs. −2.06 at 3 h) in the *high-* compared to the *low-dose* group (*F* = 7.22, *p* = 0.01). State anxiety significantly increased after endotoxin administration in both groups (*low-dose*: *F* = 4.13, *p*<0.05; *high-dose*: *F* = 6.47, *p*<0.01).

### Memory

**Figure 6:**
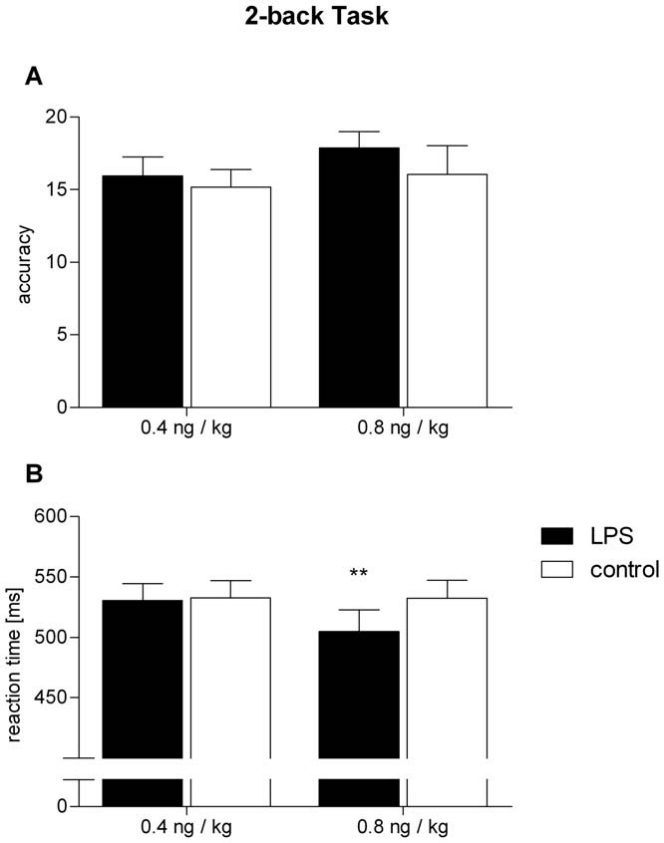
Working memory performance. Accuracy (A) and reaction time (B) in the computerized n-back working memory task (2-back version) 2 h after administration of endotoxin (black bars) or saline (white bars) within the same individuals, respectively. The *low-dose* group receiving 0.4 ng *E. coli* endotoxin per kg of body weight is represented on the left, and the *high-dose* group receiving 0.8 ng/kg on the right. The maximum possible accuracy score in (A) was 25. Data are presented as means ± SEM. Significant differences between experimental condition and respective saline control: **p<0.01 (Bonferroni post hoc test).

Working memory was assessed 2 h after LPS injection with the n-back task. Whereas the accuracy, represented by the number of correct responses minus the number of ‘false alarms’ (i.e. a response to an inadequate stimulus), remained unaffected by endotoxin in both groups (Fig. 6A; *F* = 0.20, *p*>0.05; *low-dose group*: 15.9±5.4 vs. 15.2±5.0,), there was a significant group×treatment interaction for the mean reaction time (Fig. 6B; *F* = 4.72, *p*<0.05), which was significantly reduced by 27.6 ms after endotoxin administration exclusively in the *high-dose* group (504.9±71.5 ms) compared to the placebo condition (532.5±59.3 ms; *t* = 3.21, *p*<0.01; *low dose group*: 530.7±58.2 vs. 532.6±60.3 ms).

**Figure 7:**
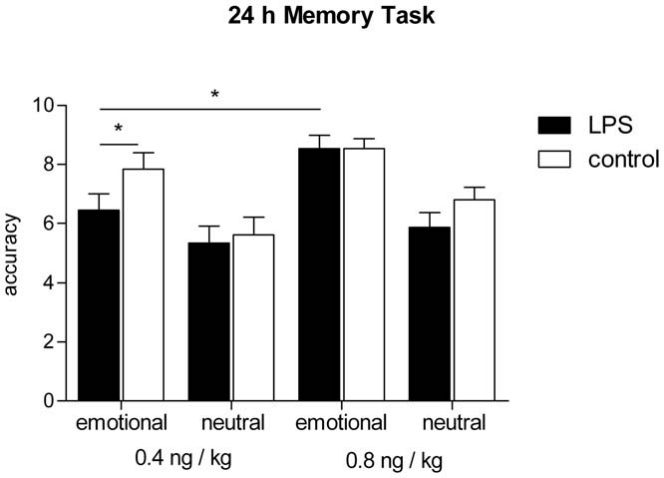
Long-term memory performance. Long-term memory performance for emotionally arousing and neutral stimuli after administration of LPS (black bars) or saline (white bars). Left panel: *low-dose* group with subjects who received 0.4 ng LPS per kg of body weight. Right panel: *high-dose* group with subjects who received 0.8 ng/kg. Data are presented as means ± SEM. Significant differences between the two groups or between experimental condition and respective saline control: *p<0.05 (Bonferroni corrected t-test). Except for the *low-dose* LPS condition, memory performance was always significantly higher for emotional than for neutral stimuli (Bonferroni corrected t-tests, not illustrated).

Long term memory performance for neutral and affective contents was analyzed with standardized stimuli of the International Affective Picture System (IAPS) (see methods section, examples given in Fig. S1). In general, subjects recognized emotional stimuli better compared to stimuli with neutral content indicated by a pronounced emotionality effect (*F* = 26.26, *p*<0.001, emotionality×treatment×group ANOVA) (Fig. 7). In addition, we observed a significant treatment effect (*F* = 7.36, *p*<0.05) due to a decreased memory performance for emotional (6.4±2.4 vs. 7.8±2.4) but not for neutral content (5.3±2.4 vs. 5.6±2.5) after endotoxin administration in the *low-dose* group (*t* = 2.88, *p*<0.05, Bonferroni corrected t-test). In contrast, a higher dose of LPS did not significantly affect memory performance neither for emotional (8.5±1.8 vs. 8.5±1.3) nor for neutral stimuli (5.9±2.0 vs. 6.8±1.7; *F* = 1.29, *p*>0.05), although there was a trend towards decreased memory performance for neutral stimuli after *high-dose* LPS treatment (*t* = 2.09, *p* = 0.10). Moreover, ANOVA did not reveal significant treatment×group, emotionality×group, or treatment×emotionality effects, however showed a significant treatment×emotionality×group interaction effect (*F* = 6.14, *p*<0.05) indicating a specific effect depending on the quality of memory content and the employed dose of LPS.

### Correlation analyses

In the *high-dose* group correlation analyses corrected for 8 comparisons revealed a negative association between changes in IL-6 levels and positive mood (*r* = −.654, *p*<0.01), calmness (*r* = −.654, *p*<0.01) as well as a positive correlation between IL-6 and state anxiety (*r* = .762, *p*<0.005). A correlation between IL-6 and mood in the *low-dose* group, however, failed to reach the adjusted alpha level. In the *low-dose* group mood changes were negatively correlated with the changes in plasma cortisol (*r* = −.739, *p*<0.005) and saliva cortisol (*r* = −.652, *p*<0.01), which was also seen in the *high-dose* group but failed to reach statistical significance after alpha correction. Furthermore, changes in plasma cortisol were associated with changes in state anxiety within the *low-dose* group (*r* = .715, *p*<0.005).

## Discussion

We employed a human endotoxemia model in healthy subjects and analyzed the effects on circulating cytokines, neuroendocrine parameters as well as mood and memory performance by either administering lower (0.4 ng/kg) or higher (0.8 ng/kg) doses of LPS. We observed significant, mainly dose-dependent, increases in body temperature, heart rate, levels of pro- (IL-6, TNF-α) and anti-inflammatory (IL-10, IL-1ra) cytokines, cortisol, and norepinephrine. Significant, dose-related increases in negative mood and anxiety confirmed that the transient inflammatory response was sensed and processed by the CNS. LPS administration did not affect accuracy in working memory, however induced a significant improvement in reaction time in the *high-dose* LPS condition. In contrast, long term-memory for emotional but not for neutral stimuli was significantly impaired by the administration of *low-dose* LPS, whereas in the *high-dose* LPS condition memory remained unaffected.

The alterations in cytokines and cortisol levels observed in this study resemble those reported in other studies employing comparable amounts (0.4 ng/kg) of intravenously administered LPS [36]. In addition, higher LPS concentration (0.8 ng/kg) further increased TNF-α, IL-10 and IL-1ra plasma concentrations, but not plasma levels of IL-6, cortisol, or norepinephrine. We also observed a pronounced and dose-dependent impact of LPS on self-reported mood with increased anxiety levels and impaired calmness and alertness. Studies in experimental animals together with clinical observations in humans suggest a cytokine-mediated modulation of memory functions as a consequence of acute peripheral inflammatory processes [3], [37]. However, data in humans so far reported inconsistent results of experimental endotoxemia with a significant improvement [24] or no effect on working memory [25], [27]. These conflicting results might be either due to the different experimentally induced grades of inflammation or the neuropsychological tools (Digit Span Forward, Backward Test by Wechsler) employed in these studies to assess working memory performance. These measures might not be sensitive enough to detect small changes induced by experimental manipulations in young, healthy subjects [38], [39], [40]. Thus, in the current study we employed a computerized n-back paradigm (2-back version), a well-evaluated sensitive tool to analyze working memory functions [34], [41], [42]. We did not find any effect on accuracy in the n-back test, reflected by the number of correct responses minus the number of false reactions, neither in the *low-* nor *high-dose* LPS condition. However, administration of 0.8 ng/kg LPS significantly improved the reaction time, possibly indicating a positive effect of the peripheral inflammation on alertness and response speed rather than on memory performance. This data might also explain the inconsistent results of former studies using the Digit Span Tests by Wechsler, which does not allow analyzing these two parameters separately [43]. Cytokine-cholinergic interactions on the basis of a negative association between changes in working memory performance and alterations in acetylcholinesterase (AChE-R) cleavage after LPS stimulation in humans have been hypothesized as a possible mechanism for working memory improvement [24]. Studies in rodents and monkeys have established that acute stress rapidly impairs dorsolateral prefrontal cortex (DLPFC) functioning via noradrenergic and dopaminergic mechanisms [44]. However this effect appears to be rather short lived [45]. In humans experimental or pharmacological stress studies have reported inconsistent effects with impairments as well as enhancements being reported [42], [46], [47]. Of interest for the current experiments is a recent functional magnetic resonance (fMRI) study reporting that several hours after cortisol intake DLPFC activity during an n-back task was enhanced with a similar trend being observed at the behavioral level. These authors suggest that slow genomic effects of cortisol enhance PFC functioning [48]. In line with this conclusion are recent findings in rodents showing that acute stress enhanced working memory via an enhancement of glutamatergic neurotransmission [49].

Interestingly, the profound decrease in self-reported alertness clearly contrasts the improvement in reaction time measured with the n-back test. Although the reasons for this discrepancy remain unclear, it underlines the basic independency of these two processes and might fortify the view on sickness behavior as an adaptive response rather than a simple impairment by the inflammatory challenge [4].

In parallel to working memory functions long term memory performance was analyzed employing a picture-recognition test consisting of neutral as well as emotional stimuli. Subjects were exposed to the stimuli 3 h after LPS injection (acquisition) and were asked to recall the stimuli 24 h later (Fig. 1). Under placebo conditions, we observed improved memory performance for emotionally arousing compared to neutral stimuli, confirming earlier observations [50]. This effect reflects the modulatory role of the amygdala on hippocampus based declarative and episodic memory [51].

Administration of *low-dose* LPS 3 h before acquisition impaired memory performance for emotional but not for neutral stimuli. Surprisingly, this effect was not observed after administration of the higher LPS dose, which did not significantly affect long term memory performance neither for neutral nor for emotional stimuli.

Animal data suggest detrimental effects of acute systemic inflammation on long term memory [3], [19], [21], 22,23. However, experimental data in humans are controversial, either reporting impaired [24], [28] or unaffected declarative memory performance [25], [26], [27] after LPS administration. Since data on dose dependent effects of LPS-induced inflammation on neural responses are completely lacking, one can only hypothesize about the underlying neurobiological mechanisms responsible for these differential effects.

Two classes of soluble factors are most often discussed to modulate cognitive functions during an inflammatory reaction: cytokines and stress hormones. Under physiological conditions cytokines have a beneficial role on learning and memory, e.g. by promoting long term potentiation (LTP), neural plasticity and neural excitability [3], [52], [53]. Administered in higher doses or during acute inflammation cytokines demonstrated detrimental effects on learning and memory [3], [54], [55]. A possible explanation for this phenomenon was given by Yirmiya and Goshen, who proposed a model of adaptive down-regulation of neural excitability to prevent potentially dangerous hyper-excitability and proneness to errors by the “price” of impairments of learning and memory [3]. Thus, the impaired memory performance following *low-dose* LPS administration might reflect this down-regulation of neural excitability. The abrogation of this impairment in the *high-dose* LPS group might either reflect a balanced state between cytokine induced increased neural excitability and the counter-acting regulatory mechanisms, or might be due to increased stress exposure: Behavioral and pharmacological studies in humans showed enhanced long-term memory consolidation for emotional stimuli after stress or glucocorticoid (GC) treatment directly before or after encoding, while impairing the consolidation of neutral material [56], [57], [58]. Thus, the absence of a memory impairment of emotional stimuli in the *high-dose* LPS group together with a trend towards impaired memory performance for neutral stimuli might be due to actions of the higher and more sustained cortisol response observed after *high-dose* LPS administration. In addition, greater effects on mood and anxiety than after *low-dose* stimulation, together with reduced reaction time in the working memory test also might indicate an elevated stress reaction and an “alarmed state” of the organism probably going along with increased attention.

However, the exact nature of possible compensatory mechanisms and in particular the question whether the compensatory effect on memory performance is paralleled by or due to increased attention need to be addressed in future studies.

The observation that in this study only memory performance for emotional stimuli was impaired by LPS administration together with the pronounced effects on mood and anxiety indicate that predominantly limbic structures like the amygdala are affected by an acute peripheral inflammatory response. This hypothesis is supported by recent work in rodents, which demonstrated enhanced neural activity in the amygdala after peripheral LPS-administration [59], [60]. However, a recent fMRI study also employing endotoxin application in humans did not report changes of neural activations in the amygdala [61]. In this study, we focused on LPS-effects on the acquisition phase of the memory process with differential and dose-dependent effects on short and long term memory. However, the distinct effects of peripheral transient inflammatory responses on memory processes such as consolidation and retrieval are still unknown and might, as a target for future studies, help to further complete the puzzle of immune-to-brain communication.

Correlation analyses revealed strong associations between the increases in anxiety and negative mood and circulating IL-6 levels in the *high-dose* and concentrations of cortisol in the *low-dose* LPS group. Associations between circulating IL-6 levels and negative mood have been reported previously [28], [62] and IL-6 frequently has been discussed as a potential modulator of mood during sickness behavior and even depression and other neuropsychological diseases [2], [3], [9]. HPA system activation with increased levels of cortisol and corticotropin releasing hormone have also been suggested to play a role in depressive symptoms and in mediating inflammatory effects on mood and anxiety [6], [28], [37], [63]. Our results confirm earlier observations which suggested both circulating cytokines as well as cortisol to be involved in mediating the effects on emotions [28]. The data in the current study show an association between cortisol and mood parameters at a lower grade of inflammation in contrast to a more pronounced inflammatory effect where mood parameters are predominantly associated to IL-6 levels.

In summary, LPS administration in healthy male subjects induced a transient, dose-dependent inflammatory response characterized by increases in body temperature and heart-rate, plasma concentrations of pro- and anti-inflammatory cytokines, cortisol, and norepinephrine. In parallel, we observed dose-dependent increases in negative mood and anxiety. There was a significant improvement in reaction times during a working memory performance task after *high-dose* LPS stimulation, whereas accuracy in the same test remained unaffected under both conditions. The innate immune response resulted in impaired memory performance for emotionally arousing material within the *low-dose* LPS condition. This study demonstrates that sub-septic systemic inflammation in humans along with the release of pro- and anti-inflammatory cytokines does dose-dependently affect neurobehavioral functions in humans reflecting an adaptive response as a consequence of a refined immune-to-brain communication.

## Supporting Information

Figure S1 **Stimuli examples for long-term memory task.** Emotional stimuli rated with high arousal and low valence score (A) and neutral stimuli rated with low arousal and medium valence score (B).
(TIF)

Figure S2 **Illustration of the n-back task paradigm.** After a training phase a consecutive sequence of 155 letters was presented on a computer screen with a presentation time of 1 second for each letter and a 30 seconds break after every 31 letters. The participants were instructed to press a button whenever the letter currently presented was identical to the penultimate. Stimuli were presented 25 times throughout the whole sequence in a randomized manner. Reaction time and the numbers of correct reactions (cr) and false alarms (fa) were assessed and accuracy ( = cr - fa) was calculated.
(TIF)
