# Supplementary figures and images for: Dose-Dependent Effects of Endotoxin on Neurobehavioral Functions in Humans
Source: PLoS One. 2011 Dec 2;6(12):e28330. doi: 10.1371/journal.pone.0028330 (PMC3229570; doi:10.1371/journal.pone.0028330)

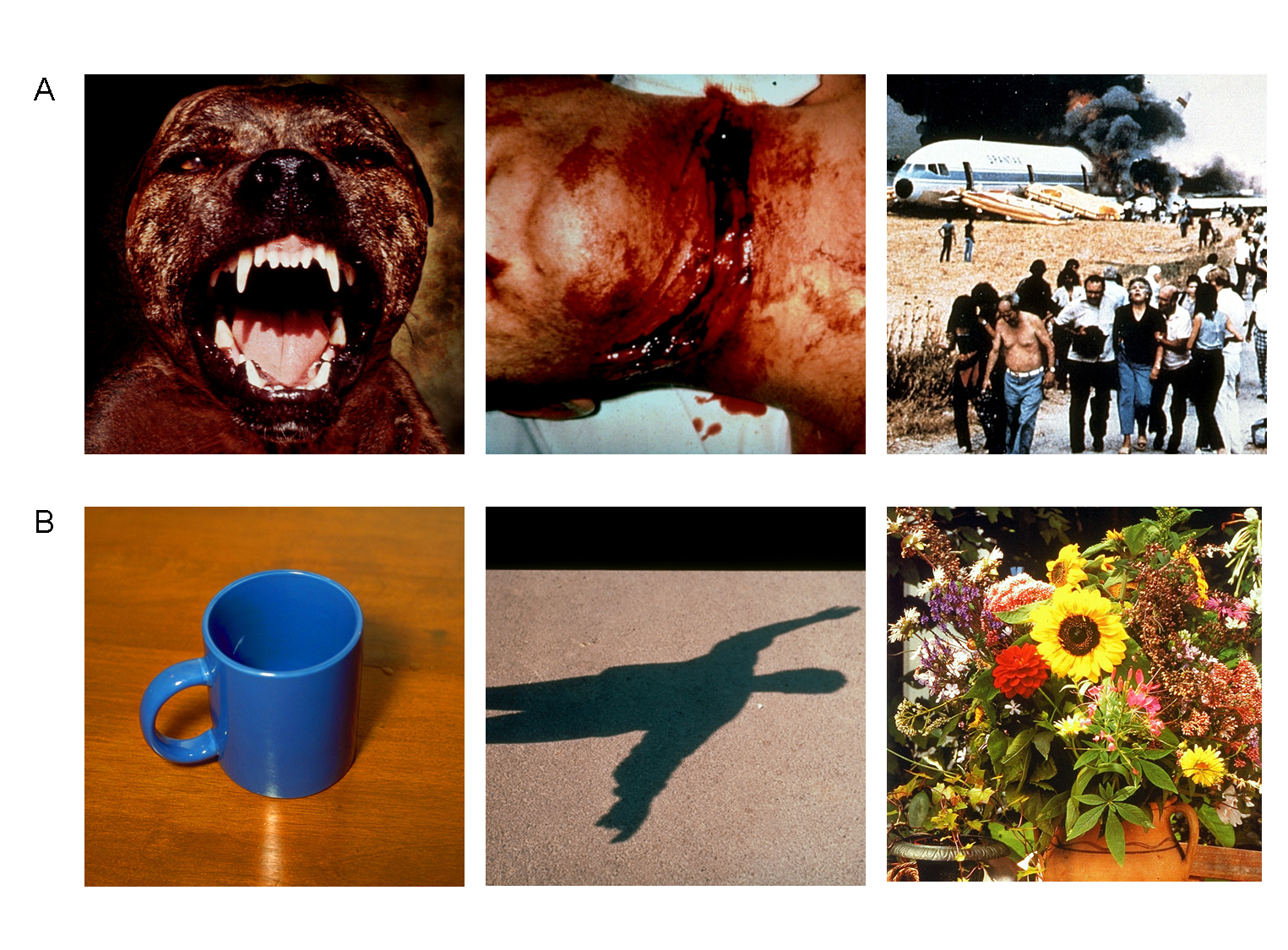

Supplement: Figure S1 — Stimuli examples for long-term memory task. Emotional stimuli rated with high arousal and low valence score (A) and neutral stimuli rated with low arousal and medium valence score (B). (TIF) [file pone.0028330.s001.tif]

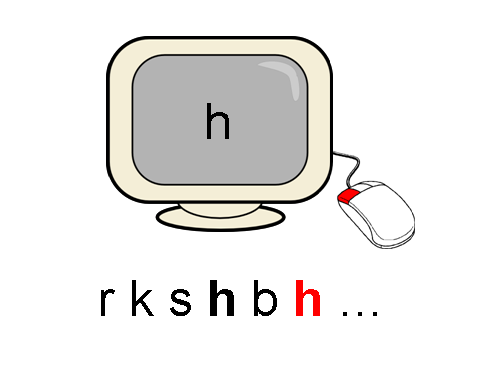

Supplement: Figure S2 — Illustration of the n-back task paradigm. After a training phase a consecutive sequence of 155 letters was presented on a computer screen with a presentation time of 1 second for each letter and a 30 seconds break after every 31 letters. The participants were instructed to press a button whenever the letter currently presented was identical to the penultimate. Stimuli were presented 25 times throughout the whole sequence in a randomized manner. Reaction time and the numbers of correct reactions (cr) and false alarms (fa) were assessed and accuracy ( = cr - fa) was calculated. (TIF) [file pone.0028330.s002.tif]
